# Long-term glycemic variability and risk of peripheral artery disease: a systematic review and meta-analysis of cohort studies

**DOI:** 10.7717/peerj.21686

**Published:** 2026-09-08

**Authors:** Jie Xia, Qionghui Peng, Lei Chen, Chiqiong Liu, Ting Zhu

**Affiliations:** School of Medicine, Hunan Polytechnic of Environment and Biology, Hengyang, China

**Keywords:** Glycemic variability, Hyperglycemia, Meta-analysis, Peripheral artery disease, Risk factor

## Abstract

**Background:**

A systematic review and meta-analysis to evaluate the impact of long-term glucose variability (GV) on the risk of developing peripheral artery disease (PAD).

**Methods:**

The protocol was prospectively registered in PROSPERO (ID: CRD420251148763). Relevant longitudinal studies were identified through comprehensive searches of PubMed, Embase, and Web of Science. The primary outcome was the risk ratio (RR) of PAD comparing participants with high versus low GV. Summary effect sizes were calculated using a random-effects model to account for between-study heterogeneity.

**Results:**

Eleven cohorts were included. Higher GV showed a positive association with PAD risk (RR: 1.42; 95% CI [1.21–1.66] *p* < 0.001), although substantial heterogeneity was present (*I*^2^ = 91%). This association was consistent across subgroups defined by region (Asian *vs.* Western), study design, diabetic status, GV metrics, PAD diagnostic methods, and adjustment for HbA1c (all *p* for subgroup differences > 0.05), except for follow-up duration. Studies with follow-up < 8 years showed a stronger association than those with ≥ 8 years (RR: 1.64 *vs.* 1.19; *p* for subgroup difference = 0.006).

**Conclusions:**

Elevated long-term GV appears to be associated with an increased risk of PAD. However, substantial heterogeneity across studies suggests that the magnitude of this association should be interpreted with caution.

## Introduction

Peripheral artery disease (PAD) represents a common manifestation of systemic atherosclerosis, characterized by the progressive narrowing or occlusion of arteries in the lower extremities (Firnhaber & Powell, 2019; Houghton et al., 2024). Globally, over 200 million individuals are affected by PAD, with prevalence increasing alongside aging, diabetes, and other cardiovascular conditions (Mandaglio-Collados, Marín & Rivera-Caravaca, 2023; Martin et al., 2025). PAD imposes substantial health burdens, including disability, reduced quality of life, elevated risk of cardiovascular events, and, in advanced stages, limb amputation (Criqui et al., 2021). Major risk factors for PAD include cigarette smoking, hypertension, dyslipidemia, chronic kidney disease, and diabetes mellitus (DM) (Aboyans et al., 2018; Song et al., 2019). Among these, hyperglycemia and diabetes are particularly influential; epidemiological data indicate that individuals with type 2 diabetes have a two- to four-fold higher risk of developing PAD compared to non-diabetic counterparts (Aboyans et al., 2018; Song et al., 2019; Verma et al., 2024). Moreover, patients with both PAD and diabetes experience poorer clinical outcomes, such as increased rates of cardiovascular complications and limb-related adverse events (Verma et al., 2024), underscoring the importance of identifying glycemia-related risk determinants beyond mean glucose levels to improve PAD prevention and management.

Long-term glucose variability (GV) refers to fluctuations in blood glucose or glycated hemoglobin (HbA1c) levels over months to years, reflecting the stability of glycemic control (Ravi et al., 2021). Various indices quantify GV, including the coefficient of variation (CV), standard deviation (SD), average real variability (ARV), variability independent of the mean (VIM), and composite measures such as the HbA1c variability score (VS) (Ajjan, 2024; Monnier et al., 2023). These parameters capture the amplitude and frequency of long-term fluctuations in fasting plasma glucose (FPG) or HbA1c across repeated measurements (Ajjan, 2024; Monnier et al., 2023). Unlike mean glycemic levels, which represent average glucose exposure, GV captures intermittent metabolic stress that may exacerbate vascular injury through mechanisms such as oxidative stress, endothelial dysfunction, and promotion of pro-inflammatory and pro-thrombotic states (Belli et al., 2023; Wang & Cao, 2025). Increasing evidence suggests that GV is also associated with a range of macrovascular and microvascular complications, including cardiovascular disease, nephropathy, retinopathy, and neuropathy (Belli et al., 2023; Emanuelsson et al., 2020). However, studies investigating its association with PAD risk have yielded inconsistent results, with some reporting significant associations and others finding null effects (Guo et al., 2022; Qu et al., 2022; Sartore et al., 2023). In light of the conflicting evidence and the clinical importance of PAD, we performed a systematic review and meta-analysis of longitudinal cohort studies to clarify the relationship between long-term GV and PAD risk in adults. We further conducted subgroup and meta-regression analyses to explore potential sources of heterogeneity. Clarifying the role of GV in PAD development could inform more nuanced glycemic monitoring and management strategies aimed at reducing PAD incidence and its associated complications.

## Materials and Methods

This meta-analysis was conducted in accordance with the PRISMA 2020 statement (Page et al., 2021) and the Cochrane Handbook for Systematic Reviews and Meta-Analyses (Higgins et al., 2021), covering protocol development, data collection, statistical procedures, and reporting. The protocol was prospectively registered in PROSPERO (ID: CRD420251148763).

### Literature search

We conducted a comprehensive search of PubMed, Embase, and Web of Science to identify eligible studies. The search strategy combined the following term groups: (1) “HbA1c” OR “glycosylated hemoglobin” OR “glucose” OR “glycemic”; (2) “variability” OR “variation” OR “fluctuation” OR “coefficient of variation” OR “standard variation”; (3) “peripheral artery disease” OR “peripheral arterial disease” OR “peripheral vascular disease” OR “critical limb ischemia” OR “intermittent claudication” OR “peripheral artery obstructive disease” OR “lower-extremity artery disease” OR “Buergers disease” OR “PAD” OR “PAOD” OR “LEAD” OR “CLI”; and (4) “cohort” OR “longitudinal” OR “prospective” OR “retrospective” OR “prospectively” OR “retrospectively” OR “followed” OR “follow-up” OR “incidence” OR “risk.” Only full-text, peer-reviewed articles in English and conducted in humans were eligible. We also manually checked the references of relevant reviews and original reports for further eligible studies. The search covered all records from database inception to April 16, 2026. The detailed search strategy for each database is shown in Supplemental File S1. Two authors (Qionghui Peng and Lei Chen) independently performed the Search Strategy, with disagreements resolved by discussion with the referee (Jie Xia).

### Inclusion and exclusion criteria

Study selection was guided by the PICOS framework:

Population (P): Adults aged ≥ 18 years without PAD at baseline; both diabetic and non-diabetic populations were eligible.

Intervention/Exposure (I): Participants classified as having high long-term GV at baseline, defined according to parameters and cutoffs specified in the original studies.

Comparison (C): Participants with low long-term GV at baseline.

Outcome (O): Development of PAD during follow-up, which was assessed using diagnostic approaches consistent with those applied in the primary studies.

Study design (S): Observational longitudinal studies, including cohort studies, nested case-control designs, or *post-hoc* analyses of trials.

Exclusion criteria included reviews, meta-analyses, editorials, preclinical studies, pediatric populations, studies not examining long-term GV, or those lacking PAD incidence data. Studies focusing on short-term glycemic fluctuations (*e.g.*, daily variability or continuous glucose monitoring metrics) were also excluded. Potential overlap of study populations was carefully assessed by examining data sources, recruitment periods, and study characteristics. When overlap was suspected, only one dataset was included to avoid double counting, typically prioritizing the study with the largest sample size.

### Study quality evaluation and data collection

Two investigators (Qionghui Peng and Lei Chen) independently conducted literature screening, quality assessment, and data extraction, with disagreements resolved by consensus with the corresponding author (Jie Xia). Study quality was assessed using the Newcastle-Ottawa Scale (NOS) (Wells et al., 2010), which evaluates selection, control of confounding, and outcome ascertainment. The NOS assigns scores from 1 to 9, with higher values indicating better quality; studies scoring ≥ 7 were regarded as high quality. Extracted data included study characteristics (author, year, country, design), participant details (source, sample size, age, sex, diabetes status), exposure measures (GV indices, cutoffs, number of glucose assessments), follow-up duration, outcome definitions (PAD diagnostic criteria and number of incident cases), and covariates included in adjusted analyses.

### Statistical analysis

We quantified the association between long-term GV and PAD risk by calculating risk ratios (RRs) and 95% confidence intervals (CIs), comparing participants with high *versus* low baseline GV. When a study reported multiple GV indices for the same population, a single estimate was selected to maintain statistical independence (Higgins et al., 2021). In the primary analysis, the estimate with the largest effect size was used. To address potential selection bias, sensitivity analyses were performed using (1) the smallest reported effect size, and (2) a predefined hierarchical approach, preferentially selecting HbA1c-based GV metrics when available, and otherwise FPG-based metrics. Although different GV metrics (*e.g.*, HbA1c- and FPG-based indices, as well as statistical constructs like SD, CV, and VIM) capture distinct statistical properties, they all reflect the underlying construct of long-term glycemic fluctuation, which supported their combined evaluation in the primary analysis. RRs and their standard errors were derived from reported 95% CIs or *p*-values and log-transformed to stabilize variance and normalize distributions (Higgins et al., 2021). Between-study heterogeneity was assessed using the Cochrane Q test and the *I*^2^ statistic (Higgins & Thompson, 2002), with *I*^2^ thresholds of <25%, 25–75%, and >70% representing low, moderate, and high heterogeneity, respectively. Pooled estimates were obtained with a random-effects model to account for inter-study variability (Higgins et al., 2021). In addition, 95% prediction intervals (PIs) were calculated to estimate the range of effects expected in future populations, particularly in the presence of substantial heterogeneity (Higgins et al., 2021). Sensitivity analyses were performed by sequentially omitting individual studies to test robustness (Marušić et al., 2020). In the primary analysis, one reported estimate was included per study, and an additional sensitivity analysis was also performed by selecting the smallest effect size to assess the robustness of the findings. Prespecified subgroup analyses examined whether study-level characteristics influenced results, including study region (Asian *vs.* Western), design (prospective *vs.* retrospective), diabetic status, GV parameters, mean follow-up duration, PAD diagnostic method, and adjustment for baseline or mean HbA1c. Median values of continuous variables were used as subgroup cutoffs. These subgroup analyses (including those addressing GV metric type, diabetic status, and PAD definition) were prespecified and primarily intended as sensitivity analyses to evaluate the robustness of the overall findings in the presence of exposure and outcome heterogeneity. Univariate meta-regression analyses explored whether continuous variables (mean age, proportion of men, follow-up length, NOS score) modified the association (Higgins et al., 2021). Potential publication bias was evaluated by visual inspection of funnel plots and Egger’s regression test (Egger et al., 1997). A *p*-value < 0.05 was considered statistically significant. Analyses were conducted using RevMan (version 5.3, Cochrane Collaboration, Oxford, UK) and Stata (version 17.0, StataCorp, College Station, TX, USA).

## Results

### Study selection

**Figure 1:**
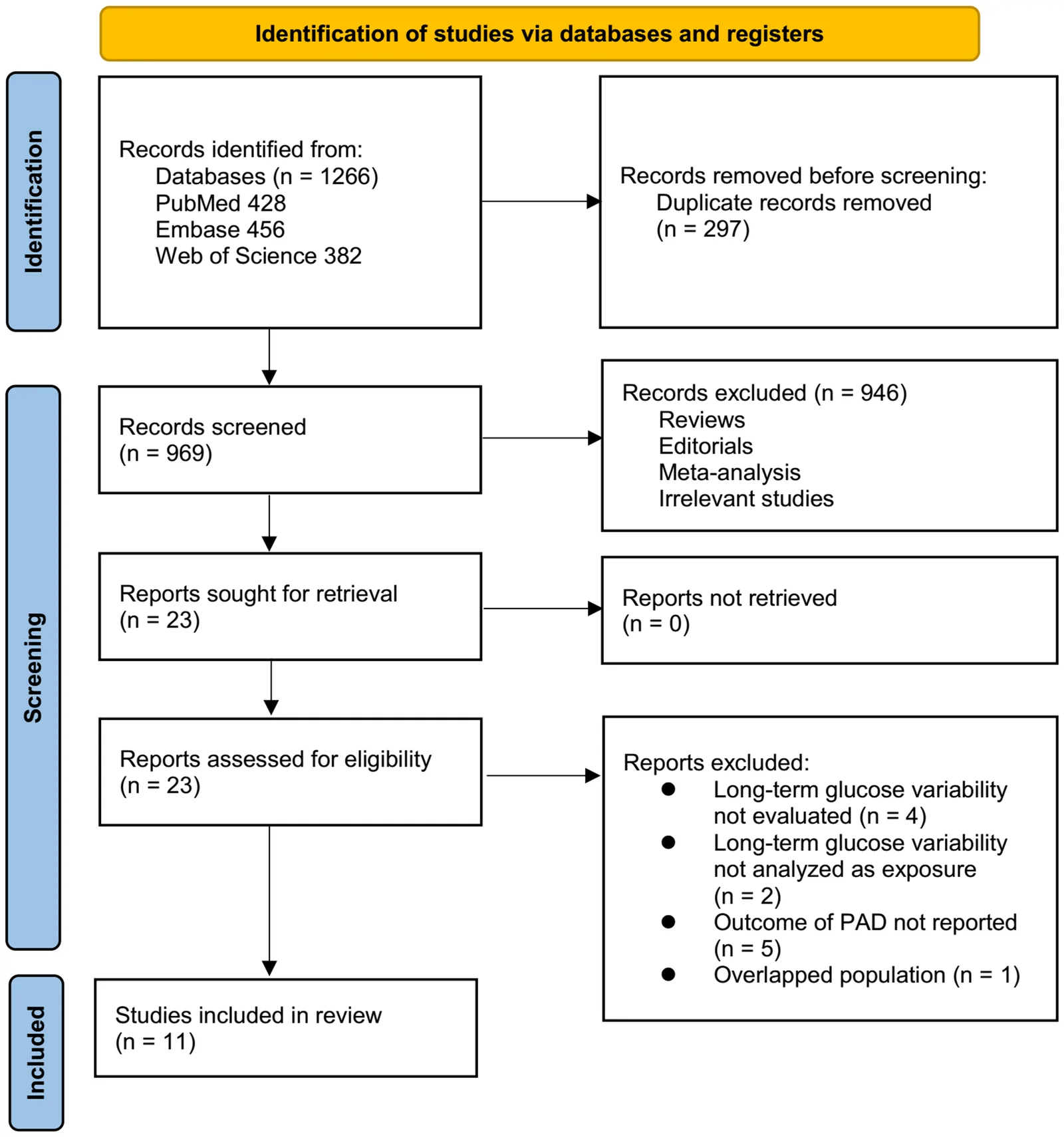
Flow diagram of the study selection process. GV, glucose variability; PAD, peripheral artery disease; PRISMA, Preferred Reporting Items for Systematic Reviews and Meta-Analyses.

The study selection process is summarized in Fig. 1. A total of 1,266 records were retrieved from the three databases, with 297 duplicates removed. Following screening of titles and abstracts, 946 articles were excluded for not fulfilling the eligibility criteria. The remaining 23 full-text papers were assessed independently by two reviewers, resulting in the exclusion of 12 studies, as detailed in Fig. 1. Consequently, 11 studies were finally included in the quantitative synthesis (Ceriello et al., 2022; Chung et al., 2022; Hsiao et al., 2023; Hsu et al., 2023; Lee, 2020b; Lee et al., 2017; Lee et al., 2020a; Shen et al., 2020; Sun et al., 2020; Tan et al., 2023; Yang et al., 2020).

### Study characteristics

Table 1 provides an overview of the principal characteristics of the included studies. A total of 11 cohorts were included, comprising three prospective cohorts (Lee et al., 2017; Shen et al., 2020; Sun et al., 2020) and eight retrospective cohorts (Ceriello et al., 2022; Chung et al., 2022; Hsiao et al., 2023; Hsu et al., 2023; Lee, 2020b; Lee et al., 2020a; Tan et al., 2023; Yang et al., 2020), published between 2017 and 2023. Studies were conducted across multiple geographic regions, including Taiwan (China), Hong Kong (China), mainland China, South Korea, Singapore, Sweden, and the United States. The study populations varied; most cohorts included diabetic populations (Ceriello et al., 2022; Hsiao et al., 2023; Hsu et al., 2023; Lee, 2020b; Lee et al., 2017; Lee et al., 2020a; Shen et al., 2020; Tan et al., 2023; Yang et al., 2020), while some included broader groups of community-derived non-diabetic participants (Chung et al., 2022; Sun et al., 2020). Altogether, the analysis incorporated 487,000 participants, with mean ages ranging from 55.5 and 67.3 years, and male proportions ranging from 44.7% to 59.3%. Various parameters were used to evaluate long-term GV, including HbA1c-SD, HbA1c-CV, HbA1c-VIM, HbA1c-ARV, HbA1c VS (HbA1c-VS), FPG-SD, FPG-CV, FPG-VIM, and FPG-ARV. Cutoffs for high GV varied across studies, with approaches such as using medians, tertiles, quartiles, or quintiles. The number of glucose measurements used to calculate GV ranged from at least 3 to an average of 12.6 per participant, and the average follow-up duration ranged from 3.8 to 19.5 years. PAD diagnosis was based on clinical criteria, including ankle-brachial index, major adverse limb events, and Doppler ultrasonography in four studies (Hsiao et al., 2023; Lee, 2020b; Shen et al., 2020; Sun et al., 2020), and International Classification of Diseases (ICD) codes in seven studies (Ceriello et al., 2022; Chung et al., 2022; Hsu et al., 2023; Lee et al., 2017; Lee et al., 2020a; Tan et al., 2023; Yang et al., 2020). During follow-up, 25,927 (5.3%) patients developed PAD. All studies adjusted for multiple covariates such as demographics, body mass index, comorbidities, cardiovascular risk factors, kidney function, lifestyle factors, and treatments, with some variation across studies.

### Quality assessment

As summarized in Table 2, NOS scores ranged from 7 to 9, indicating good methodological quality. Most studies scored well for cohort representativeness, exposure ascertainment, confirmation of outcome absence at baseline, and adjustment for key confounders (age, sex, comorbidities). Two studies (Shen et al., 2020; Sun et al., 2020) achieved the maximum score of 9, reflecting high methodological rigor. In contrast, a few studies (Hsu et al., 2023; Lee et al., 2020a; Tan et al., 2023) scored 7, mainly due to limitations in cohort representativeness and incomplete outcome assessment. All studies had adequate follow-up duration and low risk of attrition bias, supporting the reliability of the observed associations.

**Table 1:** Characteristics of the included studies.

**Study**	**Country**	**Study design**	**Population characteristics**	**No. of subjects**	**Mean age (years)**	**Men (%)**	**Parameters for GV**	**Cutoffs of GV parameters**	**Number of glucose measurements used to calculate GV**	**Mean follow-up duration (years)**	**Diagnosis of PAD**	**No. of patients with PAD**	**Variables adjusted or matched**
Lee 2017	Taiwan (China)	PC	T2DM patients	8,259	62	52	HbA1c-SD	T3:T1	At least 3	6.3	ICD codes	54	Age, sex, hypertension, retinopathy, neuropathy, mean HbA1c, triglyceride, HDL-cholesterol, eGFR, ACEI/ARB use, aspirin use, statin/fibrate use, insulin use
Lee 2020a	Hong Kong (China)	RC	T2DM patients	3,424	63	50.2	HbA1c-SD	Median	At least 3, average: 11.9 ± 4.8	10	ICD codes	NR	Age, sex, comorbidities (hypertension, IHD, stroke, HF, CKD, etc.), medications (insulin, ACEI/ARB, etc.), hypoglycemia frequency
Sun 2020	USA	PC	Community-derived non-diabetic participants	7,699	60	44.7	FPG-SD, FPG-CV, FPG-VIM, and FPG-ARV	Q4:Q1	3	19.5	ABI <0.9 and ICD codes for PAD hospitalization/revascularization	504	Age, sex, race, smoking, drinking, education, BMI, BP, lipids, baseline FPG, eGFR, CHD, HT, stroke, medication use
Shen 2020	China	PC	T2DM patients	436	58.6	59.2	HbA1c-CV, HbA1c-VIM, HbA1c-ARV	T3:T1	At least 3 within 2 years	3.8	LEAD confirmed by Doppler ultrasonography	112	Age, sex, diabetes duration, smoking status, eGFR, HDL-C, aspirin use, mean HbA1c
Yang 2020	Taiwan (China)	RC	T2DM patients	30,932	61	46.6	FPG-CV	T3:T1	At least 3 within 1 year	8.2	ICD codes	894	Age, sex, alcohol consumption, smoking, diabetes duration, antihypertensive treatment, hypoglycemic drugs, obesity, HbA1c, comorbidities
Lee 2020b	Taiwan (China)	RC	T2DM patients	4,144	66	54.2	HbA1c-SD	Median	At least 3 during follow-up	8	Composite of ABI≤0.90 and/or %MAP≥45%	770	Age, sex, CVD history, hypertension, antiplatelet use, total/HDL cholesterol, eGFR, systolic/diastolic BP, antihypertensive drugs
Chung 2022	South Korea	RC	Community-derived non-diabetic participants	152,931	55.5	59.3	FPG-CV, FPG-SD, and FPG-VIM	Q4:Q1	3–6 measurements	8.3	ICD codes	16,863	Age, sex, BMI, smoking, alcohol, exercise, income, hypertension, dyslipidemia, systolic BP, total cholesterol, history of stroke/CAD/CKD, mean FPG
Ceriello 2022	Sweden	RC	T2DM patients	101,533	64.3	55.6	HbA1c-SD	Q4:Q1	≥5 HbA1c measurements over 3 years	4.4	ICD codes	NR	Age, sex, diabetes duration, BMI, smoking, HbA1c, BP, lipids, albuminuria, eGFR, retinopathy, medications (antihypertensives, statins, aspirin), diabetes treatment
Tan 2023	Singapore	RC	DM patients	75,334	66.5	49.1	HbA1c-VS	Q5:Q1	At least 3	6	ICD codes	NR	Age, sex, ethnicity, SES (housing type), diabetes duration, BMI, smoking status, hypertension, LDL cholesterol, glucose-lowering medications
Hsu 2023	Taiwan (China)	RC	T2DM patients	45,436	67.3	52.4	FPG-CV, HbA1c-VS, HbA1c-CV, HbA1c-SD	Q4:Q1	At least 3	5.4	ICD codes	3,521	Age, sex, BMI, hypertension, CAD, baseline FPG, baseline HbA1c, eGFR, and medications (metformin, SGLT2 inhibitors, DPP4 inhibitors, GLP-1 agonists)
Hsiao 2023	Taiwan (China)	RC	T2DM patients	56,872	64	51.4	HbA1c-CV, HbA1c-SD, HbA1c-VIM, HbA1c-ARV	Q4:Q1	Average of 12.6 HbA1c measurements over 4 years	6.1	Major adverse limb events identified via ICD codes	3,209	Age, sex, BMI, smoking, comorbidities, lab data, medications, mean HbA1c, lipid profiles, blood pressure, hypoglycemia/hyperglycemia events

**Notes.**

ABI ankle-brachial index ACEI angiotensin-converting enzyme inhibitor ARV average real variability BP blood pressure BMI body mass index CAD coronary artery disease CVD cardiovascular disease CKD chronic kidney disease CHD coronary heart disease CV coefficient of variation DPP4 dipeptidyl peptidase-4 eGFR estimated glomerular filtration rate FPG fasting plasma glucose GLP-1 glucagon-like peptide-1 HbA1c hemoglobin A1c HDL high-density lipoprotein HF heart failure HT hypertension IHD ischemic heart disease ICD International Classification of Diseases LEAD lower extremity artery disease LDL low-density lipoprotein MAP mean arterial pressure NR not reported PAD peripheral artery disease PC prospective cohort Q5:Q1 the fifth vs. the first quintile Q4:Q1 the fourth vs. first quartile RC retrospective cohort SD standard deviation SES socioeconomic status SGLT2 sodium-glucose cotransporter 2 T2DM type 2 diabetes mellitus VIM variability independent of the mean VS variability score

**Table 2:** Study quality evaluation via the Newcastle–Ottawa Scale.

**Study**	**Representativeness of exposed cohort**	**Selection of non-exposed cohort**	**Ascertainment of exposure**	**Outcome absent at baseline**	**Control for age and sex**	**Control for other confounders**	**Assessment of outcome**	**Sufficient follow-up duration**	**Adequacy of cohort follow-up**	**Total score**
Lee et al., 2017	1	1	1	1	1	1	0	1	1	8
Lee et al., 2020a	0	1	1	1	1	1	0	1	1	7
Sun et al., 2020	1	1	1	1	1	1	1	1	1	9
Shen et al., 2020	1	1	1	1	1	1	1	1	1	9
Yang et al., 2020	1	1	1	1	1	1	0	1	1	8
Lee, 2020b	0	1	1	1	1	1	1	1	1	8
Chung et al., 2022	1	1	1	1	1	1	0	1	1	8
Ceriello et al., 2022	1	1	1	1	1	1	0	1	1	8
Tan et al., 2023	0	1	1	1	1	1	0	1	1	7
Hsu et al., 2023	0	1	1	1	1	1	0	1	1	7
Hsiao et al., 2023	1	1	1	1	1	1	0	1	1	8

### Meta-analysis results

**Figure 2:**
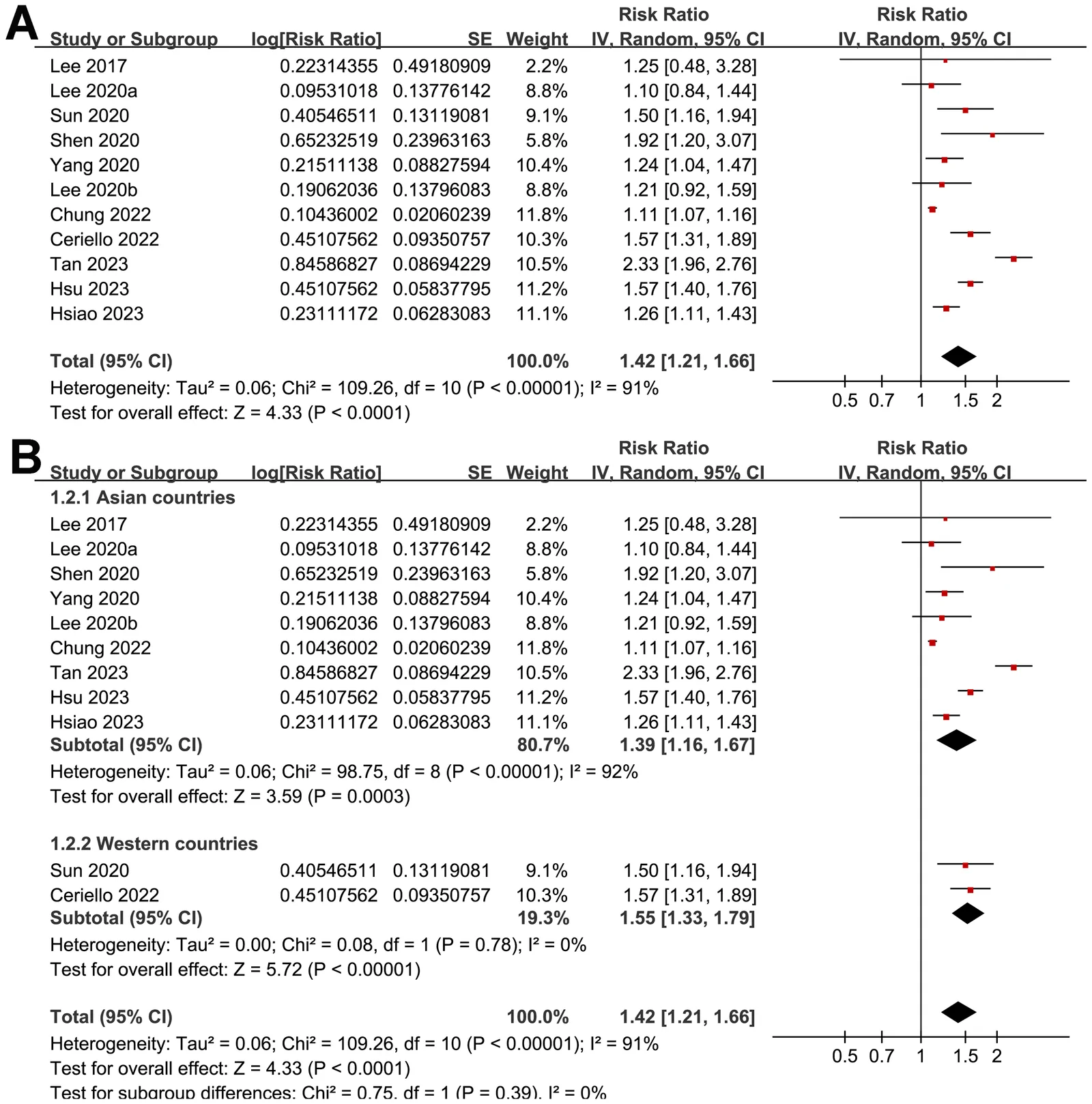
Forest plots of the association between long-term GV and risk of PAD. (A) Overall analysis; (B) Subgroup analysis by study region (Asian *vs.* Western populations). Pooled estimates were calculated using a random-effects model (inverse-variance method). Squares represent study-specific RRs, with sizes proportional to study weights, and horizontal lines indicate 95% CIs. The diamond represents the pooled RR. Heterogeneity was assessed using the *I*^2^ statistic and Cochran’s Q test. GV, glycemic variability; PAD, peripheral artery disease; RR, risk ratio; CI, confidence interval; IV, inverse variance; SE, standard error.

Pooling data across 11 cohorts (Ceriello et al., 2022; Chung et al., 2022; Hsiao et al., 2023; Hsu et al., 2023; Lee, 2020b; Lee et al., 2017; Lee et al., 2020a; Shen et al., 2020; Sun et al., 2020; Tan et al., 2023; Yang et al., 2020), elevated long-term GV was associated with a significantly increased risk of PAD (RR: 1.42, 95% CI [1.21–1.66]; *p* < 0.001; *I*^2^ = 91%; Fig. 2A), with a 95% PI of 1.03–1.95, indicating variability in the magnitude of the association across studies. Sequential exclusion of individual studies did not materially alter the findings, with pooled RRs spanning 1.33–1.47 (all *p* < 0.05). A sensitivity analysis using the lowest reported effect size for studies with multiple GV metrics yielded consistent results with the primary analysis (RR: 1.33, 95% CI [1.16–1.53]; *p* < 0.001; *I*^2^ = 88%; Fig. S1), indicating that the association was not materially influenced by the choice of GV metric.

### Subgroup analyses

**Figure 3:**
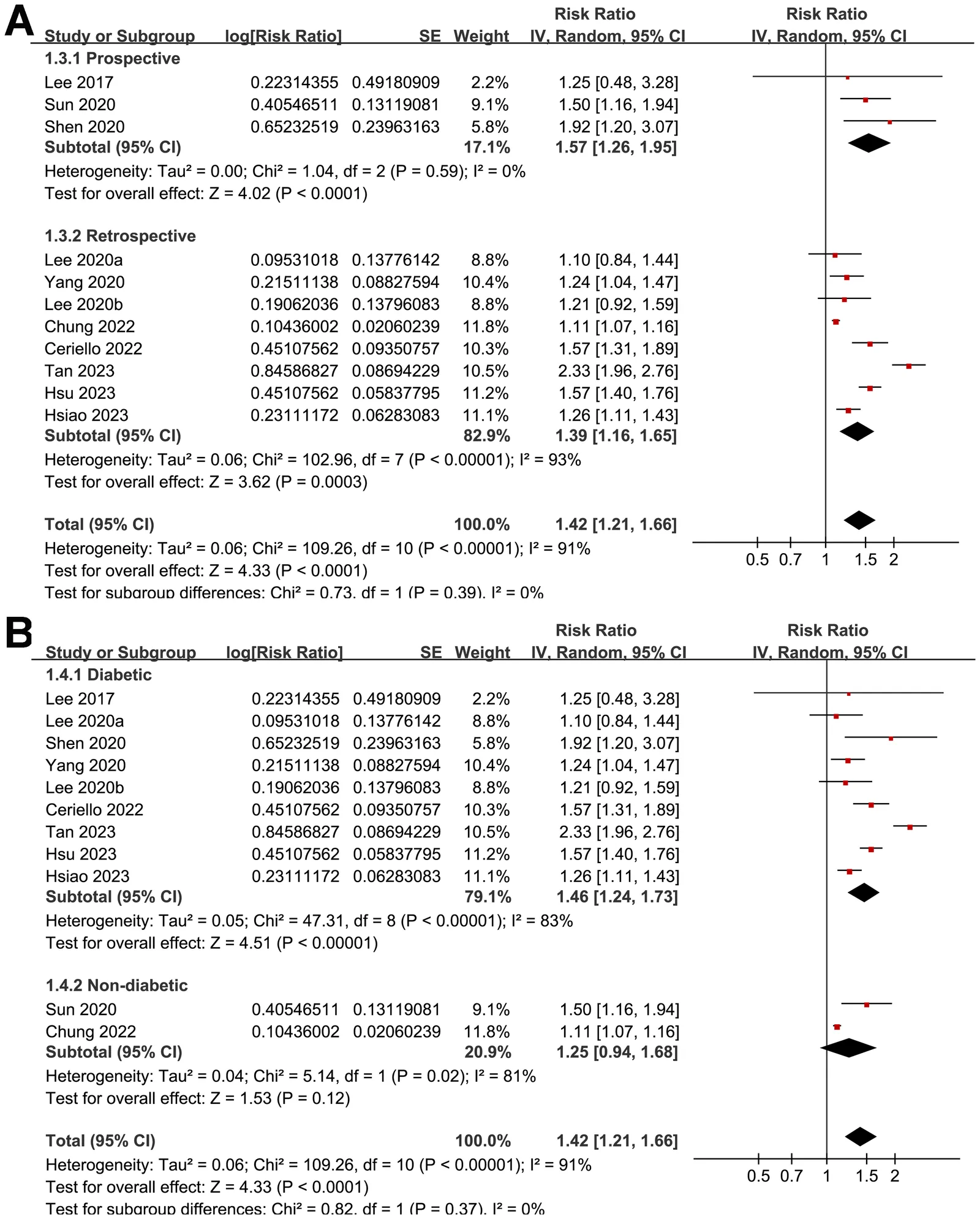
Forest plots of subgroup analyses for the association between long-term GV and risk of PAD. (A) Subgroup analysis by study design (prospective *vs.* retrospective cohorts); (B) Subgroup analysis by diabetic status (diabetic *vs.* non-diabetic populations). Pooled estimates were calculated using a random-effects model (inverse-variance method). Squares represent study-specific RRs, with sizes proportional to study weights, and horizontal lines indicate 95% CIs. The diamond represents the pooled RR. Heterogeneity was assessed using the *I*^2^ statistic and Cochran’s Q test. CI, confidence interval; GV, glycemic variability; PAD, peripheral artery disease; RR, risk ratio.

**Figure 4:**
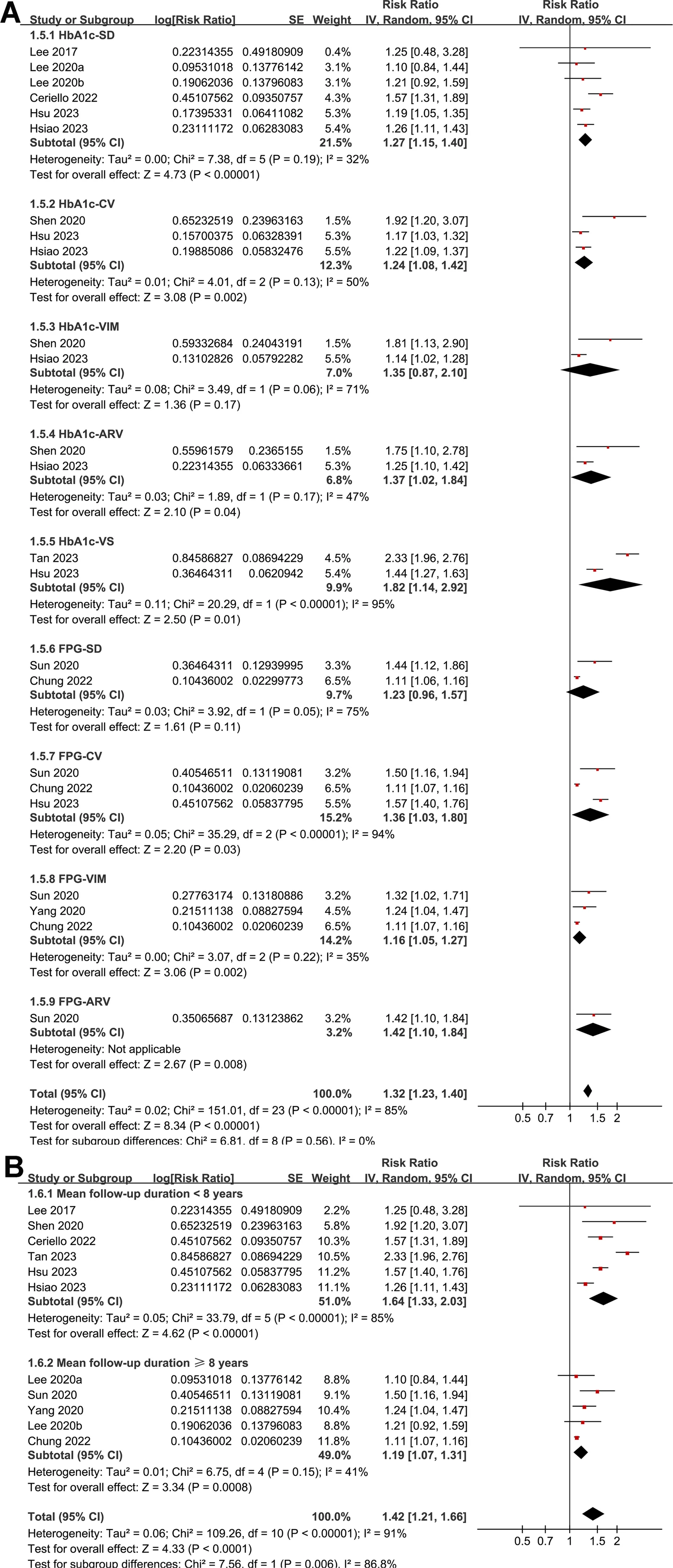
Forest plots of subgroup analyses for the association between long-term glycemic variability and risk of PAD. (A) Subgroup analysis stratified by type of GV metrics, including HbA1c-based indices (*e.g.*, SD, CV, VIM, ARV, VS) and FPG-based indices (*e.g.*, SD, CV, VIM, ARV); (B) Subgroup analysis by follow-up duration (< 8 years *vs.* ≥ 8 years). Pooled estimates were calculated using a random-effects model (inverse-variance method). Squares represent study-specific RRs, with sizes proportional to study weights, and horizontal lines indicate 95% CIs. The diamond represents the pooled RR. Heterogeneity was assessed using the *I*^2^ statistic and Cochran’s Q test. ARV, average real variability; CI, confidence interval; CV, coefficient of variation; FPG, fasting plasma glucose; GV, glycemic variability; HbA1c, glycated hemoglobin; PAD, peripheral artery disease; RR, risk ratio; SD, standard deviation; VIM, variability independent of the mean; VS, variability score.

**Figure 5:**
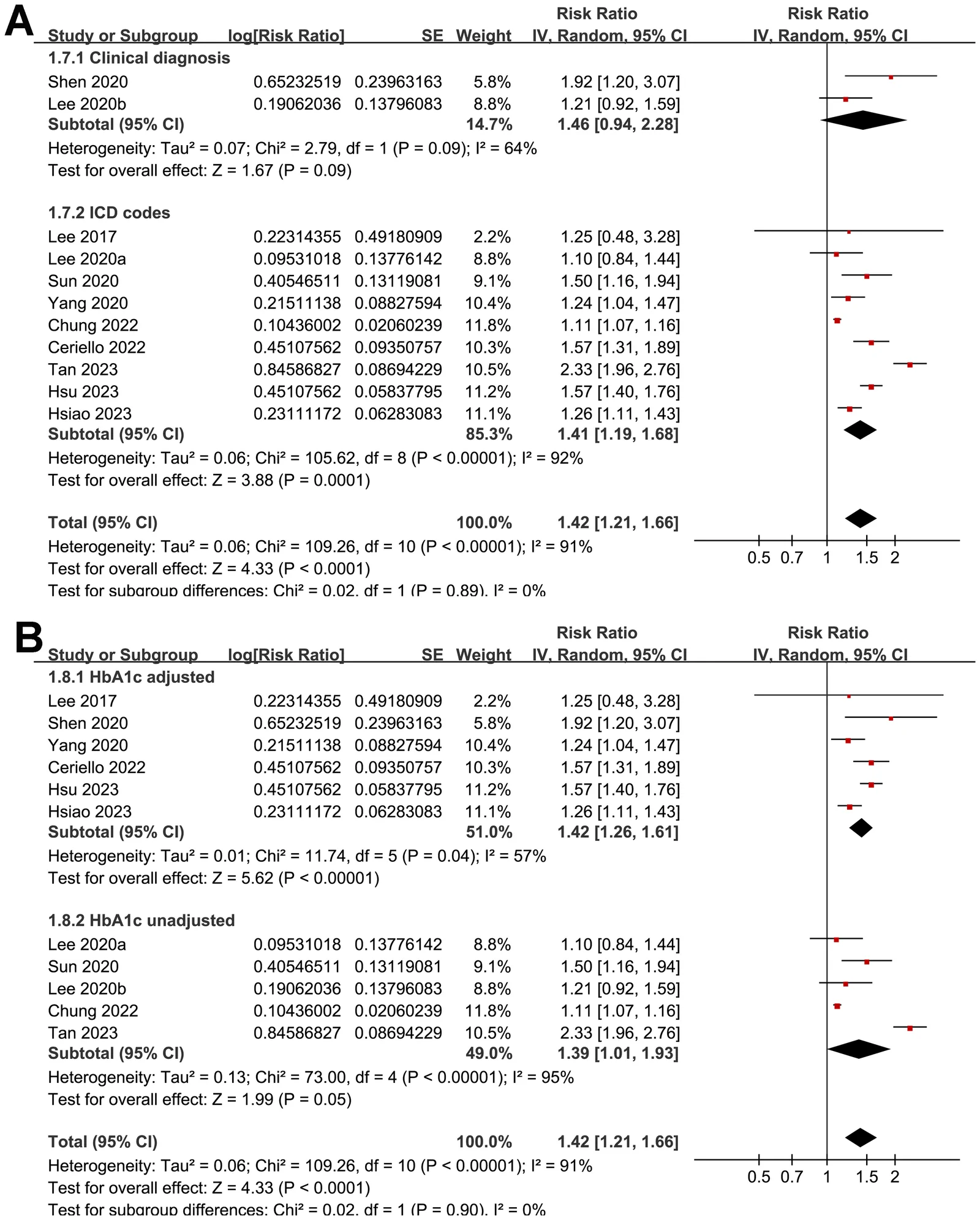
Forest plots of subgroup analyses for the association between long-term glycemic variability and risk of PAD. (A) Subgroup analysis by PAD diagnostic method, including clinical assessments (*e.g.*, ankle-brachial index (ABI), Doppler ultrasonography, or major adverse limb events) and administrative definitions based on ICD codes; (B) Subgroup analysis according to adjustment for baseline or mean HbA1c (adjusted *vs.* not adjusted). Pooled estimates were calculated using a random-effects model (inverse-variance method). Squares represent study-specific RRs, with sizes proportional to study weights, and horizontal lines indicate 95% CIs. The diamond represents the pooled RR. Heterogeneity was assessed using the *I*^2^ statistic and Cochran’s Q test. CI, confidence interval; HbA1c, glycated hemoglobin; ICD, International Classification of Diseases; RR, risk ratio.

Subgroup analyses yielded generally consistent findings across study populations (Asian *vs.* Western: RR: 1.39 *vs.* 1.55; *p* for subgroup difference = 0.39; Fig. 2B), study designs (prospective *vs.* retrospective cohorts: RR: 1.57 *vs.* 1.39; *p* = 0.39; Fig. 3A), and diabetic status (diabetic *vs.* non-diabetic: RR: 1.46 *vs.* 1.25; *p* = 0.37; Fig. 3B). Analyses stratified by different GV parameters also produced concordant results (*p* = 0.56; Fig. 4A). Notably, studies with follow-up durations < 8 years showed a stronger association than those with ≥ 8 years (RR: 1.64 *vs.* 1.19; *p* = 0.006; Fig. 4B). Associations were comparable when stratified by PAD diagnostic method (clinical assessment *vs.* ICD coding: RR: 1.46 *vs.* 1.41; *p* = 0.89; Fig. 5A) and by adjustment for baseline or mean HbA1c (RR: 1.42 *vs.* 1.39; *p* = 0.90; Fig. 5B).

### Meta-regression analysis

Univariate meta-regression results (Table 3) showed that mean age, proportion of men, follow-up length, and NOS score did not significantly modify the association between long-term GV and PAD risk (all *p* > 0.05).

### Publication bias

**Figure 6:**
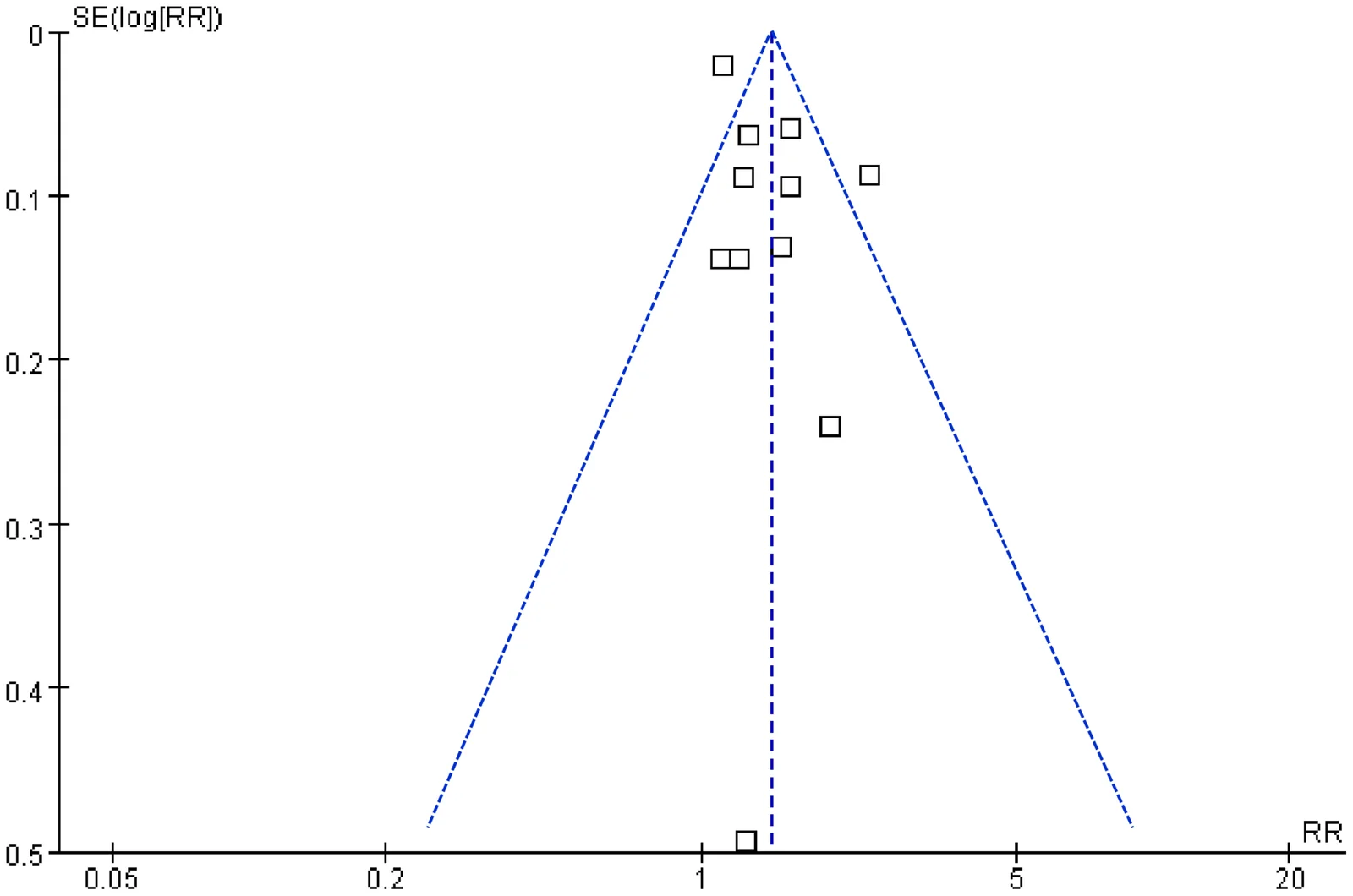
Funnel plot for assessing publication bias in the association between long-term GV and risk of PAD. Each point represents an individual study. Symmetry of the funnel plot suggests a low risk of publication bias, whereas asymmetry may indicate potential publication bias or small-study effects. GV, glycemic variability; PAD, peripheral artery disease.

Funnel plot inspection (Fig. 6) assessing the association between long-term GV and PAD suggested symmetry, indicating minimal publication bias. Egger’s regression test confirmed no significant bias (*p* = 0.55).

## Discussion

Previous meta-analyses have demonstrated associations between GV and composite cardiovascular outcomes or other complications. However, the relationship with PAD has not been specifically evaluated (Guo et al., 2022; Qu et al., 2022; Sartore et al., 2023). As PAD represents a distinct and often underdiagnosed manifestation of atherosclerosis, the present study addresses this gap by focusing on PAD incidence. In addition, we included more recent cohort data and performed subgroup and meta-regression analyses to explore potential sources of heterogeneity, providing updated and more focused evidence on this topic. This meta-analysis suggests a potential association between greater long-term GV and increased risk of PAD. However, the substantial heterogeneity indicates that the strength of this association may vary across different populations. By pooling data from eleven longitudinal cohorts comprising 487,000 participants and nearly 25,927 PAD cases, our study provides summarized evidence that the harmful effects of glycemic fluctuations extend beyond traditional glycemic indices such as mean fasting glucose or HbA1c. Importantly, the association was consistent across diverse populations, study designs, and GV metrics, reinforcing the clinical relevance of long-term GV as a risk factor for PAD.

Several biological mechanisms may explain why long-term GV contributes to PAD development. Compared with sustained hyperglycemia, oscillations in glucose levels induce greater oxidative stress, characterized by elevated reactive oxygen species and impaired endothelial function (An et al., 2023; Ceriello et al., 2008; Papachristoforou et al., 2020). Glycemic fluctuations also trigger vascular inflammation (Benhamou et al., 2014), alter nitric oxide bioavailability (Pandolfi & De Filippis, 2007), and accelerate the accumulation of advanced glycation end-products (Stirban, Gawlowski & Roden, 2014), all of which contribute to the progression of atherosclerosis in peripheral arteries. Moreover, GV may enhance platelet activation (Huang et al., 2022) and impair vascular repair mechanisms (Zhang et al., 2019), providing plausible pathways linking GV to the initiation and progression of PAD.

Substantial heterogeneity (*I*^2^ = 91%) was observed among the included studies, indicating considerable variation in the magnitude of the association between GV and PAD. This variability is likely multifactorial. First, an important source of heterogeneity relates to the pooling of different GV metrics, including HbA1c- and FPG-based indices and various statistical constructs (*e.g.*, SD, CV, and VIM). These measures capture related but not identical aspects of glycemic fluctuation and may not be fully interchangeable, introducing potential exposure heterogeneity. Similarly, combining diabetic and non-diabetic populations may involve biologically distinct baseline risks. To address these concerns, we performed prespecified subgroup analyses, which are more appropriately interpreted as sensitivity analyses, and observed generally consistent associations across GV metric types, diabetic status, and PAD definitions. However, the limited number of studies within individual strata precluded sufficiently powered separate primary analyses; therefore, residual heterogeneity and potential misclassification cannot be excluded. Second, PAD definitions varied; some studies relied on administrative ICD codes, whereas others confirmed disease using objective measures such as ABI or Doppler ultrasonography, potentially leading to differences in diagnostic accuracy and disease severity. Third, differences in population characteristics (*e.g.*, prevalence of diabetes, cardiometabolic risk factors) and geographic settings may influence study estimates. Although subgroup analyses showed no statistically significant effect modification, non-significant *p*-values do not necessarily rule out the influence of these factors and may reflect limited statistical power within subgroups. Notably, the leave-one-out sensitivity analysis demonstrated that no single study accounted for the observed heterogeneity, and meta-regression did not identify measurable modifiers, suggesting that residual confounding from unmeasured or inconsistent factors remains likely.

**Table 3:** Results of univariate meta-regression analysis.

**Variables**	**RR for the association between long-term GV and PAD**			
	**Coefficient**	**95% CI**	***p*** ** values**	**Adjusted R** ^**2**^
Mean age (years)	0.027	−0.015 to 0.069	0.18	22.0%
Men (%)	−0.007	−0.047 to 0.033	0.70	0%
Follow-up duration (years)	−0.013	−0.057 to 0.031	0.52	0%
NOS	−0.044	−0.316 to 0.227	0.72	0%

**Notes.**

Coefficients are expressed on the log risk ratio (log RR) scale.

RR risk ratio CI confidence interval GV glycemic variability NOS Newcastle–Ottawa Scale PAD peripheral artery disease

Subgroup and sensitivity analyses offer further insights. The stronger association observed in studies with follow-up durations <8 years compared with those ≥ 8 years suggests that the impact of GV on PAD risk may be more pronounced over shorter time horizons. Several explanations may account for this finding. First, reverse causality cannot be excluded, as individuals with early or subclinical vascular disease may already exhibit greater glycemic instability. Second, survivor bias may play a role, whereby individuals with higher GV and greater vascular risk are more likely to experience competing events (*e.g.*, cardiovascular mortality) during longer follow-up, thereby attenuating the observed association with PAD. Third, GV is a dynamic exposure that may change over time; baseline measurements may not adequately reflect long-term patterns, leading to potential misclassification in studies with extended follow-up. Finally, regression dilution bias may weaken observed associations over time due to within-person variability and measurement error. These factors collectively suggest that the association between GV and PAD may be influenced by follow-up duration and should be interpreted with caution. Additionally, the discrepancy between subgroup analysis and meta-regression regarding the influence of follow-up duration suggests that this finding may be influenced by arbitrary categorization or limited statistical power, and should therefore be interpreted with caution. Another key finding is that the association between GV and PAD risk remained consistent regardless of adjustment for baseline or mean HbA1c in multivariable models. This indicates that GV influences vascular outcomes independently of average glycemic levels, underscoring the importance for clinicians to monitor both aspects of glycemic control in practice (Martinez et al., 2021). The robustness of the association was confirmed by sensitivity analyses, which showed no single study disproportionately influenced the overall results. Finally, although diabetic and non-diabetic populations differ in baseline risk profiles, subgroup analyses showed broadly consistent associations, suggesting that the relationship between GV and PAD may extend across populations, although limited data in non-diabetic cohorts warrant cautious interpretation.

Several strengths characterize this work. First, the database search was thorough and up to date, spanning PubMed, Embase, and Web of Science, with screening conducted up to July 2025. Second, only longitudinal cohort studies were included, minimizing the risk of reverse causation inherent to cross-sectional data. Third, all studies adjusted for multivariable confounders, such as demographics, comorbid conditions, lifestyle factors, and treatments. These methodological strengths lend credibility to the conclusion that elevated long-term GV is a significant predictor of PAD.

Despite these strengths, certain limitations exist. First, the predominance of retrospective cohorts introduces potential bias (Kim, 2024), and most included studies were conducted in Asian populations (Chung et al., 2022; Hsiao et al., 2023; Hsu et al., 2023; Lee, 2020b; Lee et al., 2017; Lee et al., 2020a; Shen et al., 2020; Tan et al., 2023; Yang et al., 2020), which may limit generalizability. Substantial heterogeneity was observed, likely reflecting differences in population characteristics, GV metrics, and PAD definitions. In particular, diverse GV metrics (*e.g.*, SD, CV, VIM, and ARV derived from HbA1c or FPG) capture related but non-identical aspects of glycemic fluctuation; pooling these measures may have introduced exposure heterogeneity, although subgroup analyses showed broadly consistent results. In addition, most studies used categorical GV definitions with study-specific cutoffs, precluding dose–response analyses and limiting assessment of exposure–response relationships.

Another important limitation relates to the handling of multiple GV metrics within studies. In the primary analysis, we used the largest reported effect size as a pragmatic approach, which may have introduced upward bias. However, consistent results were observed in sensitivity analysis using the smallest effect size and in subgroup analyses according to GV metric type, suggesting that the overall findings are unlikely to be driven solely by selective reporting. Nevertheless, this approach is not optimal; future meta-analyses with more standardized reporting or access to individual patient data may allow more rigorous methods, such as predefined metric selection or multivariate meta-analysis.

Furthermore, PAD definitions varied across studies (clinical assessments *vs.* ICD-based diagnoses), which may have introduced outcome misclassification. Residual confounding is also likely due to inconsistent adjustment for factors such as diabetes duration, medications, and vascular comorbidities. Additionally, only studies published in English were included, which may have introduced language bias, thus limiting the generalizability of the findings. Finally, although no significant publication bias was detected, the limited number of included studies reduced the power of the funnel plot and Egger’s test; therefore, the presence of publication bias cannot be excluded.

From a clinical perspective, these findings have important implications. They suggest that stabilizing long-term glycemic control may reduce the risk of developing PAD, complementing the established goal of lowering mean glucose levels. Current diabetes management primarily targets HbA1c levels; however, our results highlight the potential value of incorporating GV assessment into routine care. Monitoring long-term GV using serial HbA1c or FPG values could help identify high-risk patients who may benefit from intensified lifestyle interventions, optimized pharmacotherapy, or closer vascular surveillance. Given that PAD is often underdiagnosed until advanced stages (Grant et al., 2023), early identification of individuals with unstable glycemic control may provide a window of opportunity for preventive strategies. Future research should build on these findings by addressing the identified limitations. Large-scale prospective cohorts with standardized definitions of GV and PAD are needed to confirm the consistency of associations across diverse populations. The collection of IPD would enable harmonization of GV metrics and more detailed analyses of interactions with factors such as diet, medication use, and genetic susceptibility. Furthermore, mechanistic studies are warranted to elucidate the biological pathways linking GV to peripheral atherosclerosis. Randomized controlled trials targeting GV reduction, potentially through novel therapies with stable glycemic profiles, could provide definitive evidence regarding causality and clinical benefit. Until such data are available, these findings should be interpreted with caution but nonetheless offer valuable guidance for clinical practice and future research priorities.

In summary, this meta-analysis suggests that higher long-term GV is associated with an increased risk of PAD. However, the magnitude of this association varies considerably across studies. Although the direction of effect was broadly consistent, substantial heterogeneity and the observational nature of the included studies limit certainty regarding the precise effect size. Therefore, GV should be considered a potential risk indicator rather than a definitive causal factor. Further large, well-designed prospective studies with standardized definitions are needed to clarify the role of GV in PAD development and to determine whether reducing GV can improve vascular outcomes.

##  Supplemental Information

10.7717/peerj.21686/supp-1 Supplemental Information 1 Forest plots for the sensitivity analysis using the RR with the lowest effect size when multiple GV indices were reported for the same population

10.7717/peerj.21686/supp-2 Supplemental Information 2 PRISMA checklist

10.7717/peerj.21686/supp-3 Supplemental Information 3 Detailed search strategy for each database

10.7717/peerj.21686/supp-4 Supplemental Information 4 Raw data extracted from the cited literature

10.7717/peerj.21686/supp-5 Supplemental Information 5 Intended audience for this article

## Additional Information and Declarations

### Data Availability

The following information was supplied regarding data availability: The raw data extracted from the included studies are presented within the manuscript as follows: All data used for statistical analyses are therefore fully available within the manuscript and its Supplementary Files, and no additional raw data are required to reproduce the results.
