# Supplementary material for: Long-term glycemic variability and risk of peripheral artery disease: a systematic review and meta-analysis of cohort studies
Source: PeerJ. 2026 Sep 8;14:e21686. doi: 10.7717/peerj.21686 (PMC13564404; doi:10.7717/peerj.21686)
Supplement: Supplementary file 2 [file peerj-14-21686-s003.docx]

**Supplemental File 1** Detailed search strategy for each database

**PubMed**

#1 Glycemic variability

("Hemoglobin A, Glycosylated"[Mesh] OR "Blood Glucose"[Mesh] OR HbA1c[tiab] OR "glycosylated hemoglobin"[tiab] OR "glycated hemoglobin"[tiab] OR glucose[tiab] OR glycemic[tiab]) AND (variability[tiab] OR variation[tiab] OR fluctuation*[tiab] OR "coefficient of variation"[tiab] OR "standard deviation"[tiab] OR CV[tiab])

#2 Peripheral artery disease

("Peripheral Arterial Disease"[Mesh] OR "Peripheral Vascular Diseases"[Mesh] OR "Intermittent Claudication"[Mesh] OR "Ischemia"[Mesh] OR "peripheral artery disease"[tiab] OR "peripheral arterial disease"[tiab] OR "peripheral vascular disease"[tiab] OR "peripheral artery obstructive disease"[tiab] OR "lower extremity arterial disease"[tiab] OR "lower-extremity artery disease"[tiab] OR "intermittent claudication"[tiab] OR "critical limb ischemia"[tiab] OR "Buerger disease"[tiab] OR PAD[tiab] OR PAOD[tiab] OR LEAD[tiab] OR CLI[tiab])

#3 Study design / outcome

("Incidence"[Mesh] OR "Risk"[Mesh] OR "Cohort Studies"[Mesh] OR "Longitudinal Studies"[Mesh] OR "Prospective Studies"[Mesh] OR "Retrospective Studies"[Mesh] OR incidence[tiab] OR risk[tiab] OR cohort[tiab] OR longitudinal[tiab] OR

prospective[tiab] OR retrospective[tiab] OR follow-up[tiab] OR followed[tiab])

#Final

#1 AND #2 AND #3

**Embase**

#1 Glycemic variability

('glycosylated hemoglobin'/exp OR 'blood glucose'/exp OR hba1c:ti,ab OR 'glycated hemoglobin':ti,ab OR glucose:ti,ab OR glycemic:ti,ab) AND (variability:ti,ab OR variation:ti,ab OR fluctuation*:ti,ab OR 'coefficient of variation':ti,ab OR 'standard deviation':ti,ab)

#2 Peripheral artery disease

('peripheral artery disease'/exp OR 'peripheral vascular disease'/exp OR 'intermittent claudication'/exp OR 'critical limb ischemia'/exp OR 'peripheral artery disease':ti,ab OR 'peripheral arterial disease':ti,ab OR 'peripheral vascular disease':ti,ab OR 'lower extremity arterial disease':ti,ab OR 'intermittent claudication':ti,ab OR 'critical limb ischemia':ti,ab OR 'buerger disease':ti,ab OR PAD:ti,ab OR PAOD:ti,ab OR LEAD:ti,ab OR CLI:ti,ab)

#3 Study design

('incidence'/exp OR 'risk'/exp OR 'cohort analysis'/exp OR 'longitudinal study'/exp OR 'prospective study'/exp OR 'retrospective study'/exp OR incidence:ti,ab OR risk:ti,ab OR cohort:ti,ab OR longitudinal:ti,ab OR prospective:ti,ab OR retrospective:ti,ab OR 'follow up':ti,ab)

#Final

#1 AND #2 AND #3

**Web of Science**

TS = ((("glycosylated hemoglobin" OR "glycated hemoglobin" OR HbA1c OR glucose OR glycemic) AND (variability OR variation OR fluctuation* OR "coefficient of variation" OR "standard deviation")) AND ("peripheral artery disease" OR "peripheral arterial disease" OR "peripheral vascular disease" OR "peripheral artery obstructive disease" OR "lower extremity arterial disease" OR "intermittent claudication" OR "critical limb ischemia" OR "Buerger disease" OR PAD OR PAOD OR LEAD OR CLI) AND (incidence OR risk OR cohort OR longitudinal OR prospective OR retrospective OR follow-up OR followed))
