# Supplementary material for: Long-term glycemic variability and risk of peripheral artery disease: a systematic review and meta-analysis of cohort studies
Source: PeerJ. 2026 Sep 8;14:e21686. doi: 10.7717/peerj.21686 (PMC13564404; doi:10.7717/peerj.21686)
Supplement: Supplementary file 3 [file peerj-14-21686-s005.docx]

**Intended audience**

This systematic review and meta-analysis is primarily intended for clinicians and researchers involved in the care of patients with diabetes and cardiovascular disease, including endocrinologists, cardiologists, vascular specialists, primary care physicians, and public health professionals. It is also relevant to epidemiologists and clinical investigators interested in glycemic variability, cardiometabolic risk stratification, and long-term vascular outcomes. In addition, the findings may inform guideline developers and healthcare policymakers by providing synthesized evidence on the potential role of long-term glucose variability in peripheral artery disease risk, thereby supporting more comprehensive approaches to glycemic assessment and prevention strategies.
